# Monitoring of Essential Levels of mental healthcare during the Covid-19 epidemic outbreak. Evidence from an Italian Real-World study

**DOI:** 10.1192/j.eurpsy.2023.867

**Published:** 2023-07-19

**Authors:** G. Caggiu, M. Monzio Compagnoni, G. Corrao, M. Franchi, A. Lora

**Affiliations:** 1 University of Milano-Bicocca; Department of Mental Health and Addiction Services, ASST Lecco, Lecco, Italy; 2 Università degli studi di Milano-Bicocca, Milano; 3 Department of Mental Health and Addiction Services, ASST Lecco, Lecco, Italy

## Abstract

**Introduction:**

Mental healthcare proved to have experienced a clear-cut reduction during the Covid-19 outbreak, and its responsiveness to patients’ health needs showed relevant declines. Moreover, the impact of the pandemic on usual outpatient healthcare has never been systematically measured with a person-level approach in analytical studies.

**Objectives:**

To assess how the access to, and the delivery of, recommended healthcare for patients with severe mental illness has changed during the Covid-19 pandemic.

**Methods:**

Data were retrieved from the HCU of Lombardy Region (Italy), and a population-based study estimated the association between the level of epidemic restrictions (free, severe, light and moderate) and the recommended healthcare provided (outcome) to patients with schizophrenic and depressive disorders. For each disorder, prevalent and incident patients in the year 2019 were identified. These patients were then observed from 1^st^ January 2020 to December 31, 2020. A Self-Controlled Case Series (SCCS) design was applied, and estimates were obtained with a conditional Poisson regression model. Adjustments for seasonality of medical services delivering were performed (SCC-RS design, with recruitment of a specific reference cohort in 2018, evaluated in 2019). The estimates were stratified according to gender, age and comorbidity profile of the patients included.

**Results:**

Patients with prevalent schizophrenic disorder were 29,516 (Prevalence Rate=35.5x10’000 inhabitants, **Image 1**), 292 with incident disorder; patients with prevalent depressive disorder were 37,764 (PR=45.4, **Image 2**), 4,349 with incident disorder. The largest reductions were observed in the rate of psychosocial interventions delivery during the period of exposure to severe restrictions (IRR: 0.35; 95% CI: 0.34 - 0.36 for patients with schizophrenic disorder and 0.49; 0.45 - 0.53 for patients with depression, **Image 3**), compared to the pre-pandemic period. For patients with incident disorder, the largest reduction concerned the delivery of psychoeducational interventions during the period of exposure to moderate restrictions (0.19; 0.06 - 0.64 for patients with schizophrenic disorder and 0.27; 0.13 - 0.55 for patients with depressive disorder), compared to the pre-pandemic period.

**Image:**

**Figure:**
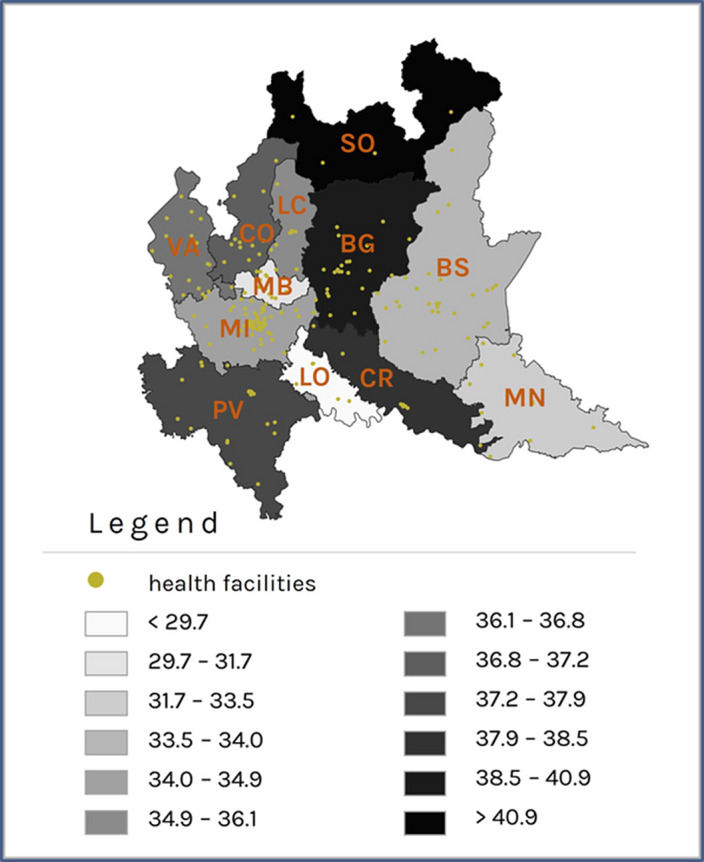

**Image 2:**

**Figure:**
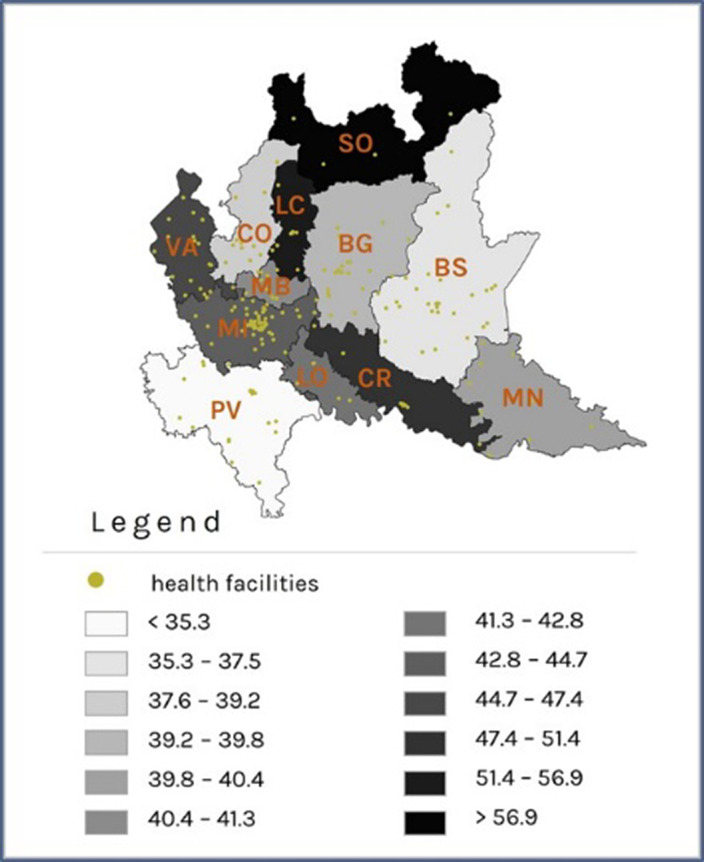

**Image 3:**

**Figure:**
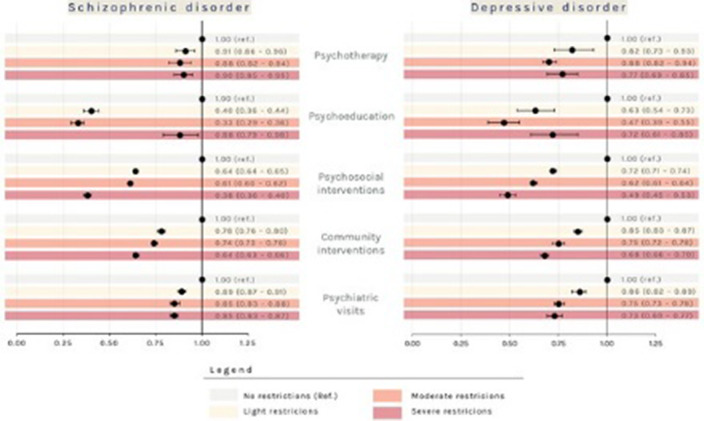

**Conclusions:**

Real-world data can be used to assess how the individual access to psychiatric recommended healthcare changed during the Covid-19 epidemic. Also, compared to the pre-pandemic period, there was a general reduction in the delivery of recommended interventions to patients with mental disorders during the pandemic period.

**Disclosure of Interest:**

None Declared

